# Developing machine learning-based models to predict intrauterine insemination (IUI) success by address modeling challenges in imbalanced data and providing modification solutions for them

**DOI:** 10.1186/s12911-022-01974-8

**Published:** 2022-09-01

**Authors:** Sajad Khodabandelu, Zahra Basirat, Sara Khaleghi, Soraya Khafri, Hussain Montazery Kordy, Masoumeh Golsorkhtabaramiri

**Affiliations:** 1 Infertility and Reproductive Health Research Center, Health Research Institute, Babol University of Medical Sciences, Babol, Iran; 2 Student Research Committee, Babol University of Medical Sciences, Babol, Iran; 3 Faculty of Electrical and Computer Engineering, Babol Noshirvani University of Technology, Babol, Iran

**Keywords:** Machine learning, Imbalanced data, Intrauterine insemination, Infertility, Cumulative live birth

## Abstract

**Background:**

This study sought to provide machine learning-based classification models to predict the success of intrauterine insemination (IUI) therapy. Additionally, we sought to illustrate the effect of models fitting with balanced data vs original data with imbalanced data labels using two different types of resampling methods. Finally, we fit models with all features against optimized feature sets using various feature selection techniques.

**Methods:**

The data for the cross-sectional study were collected from 546 infertile couples with IUI at the Fatemehzahra Infertility Research Center, Babol, North of Iran. Logistic regression (LR), support vector classification, random forest, Extreme Gradient Boosting (XGBoost) and, Stacking generalization (Stack) as the machine learning classifiers were used to predict IUI success by Python v3.7. We employed the Smote-Tomek (Stomek) and Smote-ENN (SENN) resampling methods to address the imbalance problem in the original dataset. Furthermore, to increase the performance of the models, mutual information classification (MIC-FS), genetic algorithm (GA-FS), and random forest (RF-FS) were used to select the ideal feature sets for model development.

**Results:**

In this study, 28% of patients undergoing IUI treatment obtained a successful pregnancy. Also, the average age of women and men was 24.98 and 29.85 years, respectively. The calibration plot in this study for IUI success prediction by machine learning models showed that between feature selection methods, the RF-FS, and among the datasets used to fit the models, the balanced dataset with the Stomek method had well-calibrating predictions than other methods. Finally, the brier scores for the LR, SVC, RF, XGBoost, and Stack models that were fitted utilizing the Stomek dataset and the chosen feature set using the Random Forest technique obtained equal to 0.202, 0.183, 0.158, 0.129, and 0.134, respectively. It showed duration of infertility, male and female age, sperm concentration, and sperm motility grading score as the most predictable factors in IUI success.

**Conclusion:**

The results of this study with the XGBoost prediction model can be used to foretell the individual success of IUI for each couple before initiating therapy.

**Supplementary Information:**

The online version contains supplementary material available at 10.1186/s12911-022-01974-8.

## Background

Every year more than seven million couples seek treatment for infertility, a disease that affects around 15% of couples worldwide [1]. Unsuccessful clinical pregnancy after 12 months of regular sexual intercourse is considered infertility [2, 3]. Nowadays, infertile couples rely on sophisticated laboratory technology to conceive, and various methods are used, from less aggressive to more aggressive, to help infertile couples. Due to the availability, cost-effectiveness, and low invasiveness of the IUI method compared to other methods such as in vitro fertilization (IVF), it is one of the first-line treatment proposals for infertile couples [4]. Intrauterine insemination (IUI) is an assisted reproductive therapy that places a sample of processed semen into the uterine cavity. Intrauterine insemination (IUI) is an assisted reproductive therapy that inserts a processed semen sample into the uterine cavity to increase the chance of more motile sperm entering the upper female reproductive tract.

Patients seeking Intra-Uterine Sperm Insemination (IUI) want a chance to succeed in their treatment. IUI combined with controlled ovarian hyperstimulation (COH) is a viable approach, with pregnancy rates ranging from 10 to 33% each cycle [5]. However, this method cannot guarantee a pregnancy despite the mentioned advantages. It may even lead to complications such as Ovarian hyperstimulation syndrome (OHSS), multiple pregnancies, and the risk of ovarian cancer. OHSS, with a variable prevalence of 3% to 23%, is an iatrogenic and one of the most frightening complications of ovarian stimulation [6]. This complication also exists for other treatment methods such as IVF and intracytoplasmic injection (ICSI). According to annual statistics, this is even though 1.5 million cycles of assisted reproductive technology (ART), including the three mentioned treatment methods, are performed worldwide [7]. Consequently, evidence-based tools for the probability of successful live birth before IUI treatment are needed to aid in counseling patients in clinical practice.

Researchers and experts in this field have made many efforts to solve this challenge. However, the statistical models used to predict the success of IUI have not yet been able to answer this challenge practically. Most past studies to create predictive models have focused more on the factors affecting pregnancy and not on developing new models. Logistic and Cox regression are the most used models in this field [8, 9]. However, traditional models such as the mentioned models need to make certain assumptions in the data set to fit and be valid and also cannot take advantage of interrelationships between predictors and combinations of factors that are not individually significant discriminators [10, 11]. On the other hand, today, machine learning methods are increasingly used to improve prediction for clinical decision-making [12].

Machine learning models are one of the most suitable approaches since they do not impose any basic assumptions on data distribution and may handle the mentioned issues in traditional models. Furthermore, there are no constraints on the functional structure of the connection between independent and dependent variables. Another advantage of machine learning is that the data is evaluated implicitly. As a result, even if a portion of the machine learning framework is missing or malfunctions, the correct answer can still be found. Machine learning generalizability also helps the model respond properly to an untrained new observation [13–15].

In most of the previous studies, the influential features have been selected through the weight or p-value of the fitted model. There has been no focus on the feature selection methods to choose the optimal set of features for fitting the model. This process will lead to two fundamental problems. First, the researcher may not have included effective features in the model, and second, not choosing the optimal and effective set to fit the model may reduce the accuracy and efficiency of the final model [8]. The female age, duration of infertility, sperm quality, and the number of follicles on the day of Human Chorionic Gonadotropin (HCG) injection are all crucial factors in the IUI success [16, 17].

Furthermore, one of the issues seen in previous studies is the lack of consensus on using model evaluation criteria and how to interpret them [9]. Nonetheless, the use of the AUC index as the area under the ROC curve in the evaluating models is increasing, even though this criterion has limitations.

Beyond these, due to the relatively low success rate in ART methods, we will encounter imbalanced data in classes, which may be effective in fitting and evaluating prediction models.

Therefore, we pursue three goals in this study:

- Creating a model based on machine learning to predict the success of the IUI method and using different evaluation criteria to measure the efficiency of the models.
- Using different methods to select the optimal set of features for model development.
- And also, data balancing methods investigate the impact of data imbalance in the development and efficiency of predicting models.

## Methods

### Data collection

Our cross-sectional study included the data from 546 infertile couples who had IUI in Mehrgan and Fatemeh Zahra Infertility Centers of Babol University of Medical Sciences, Mazandaran Province, North of Iran. All couples provided an entire medical history, and all female participants received hysterosalpingography, a clinical laboratory assessment, and a thorough physical examination. Following the Nordic Society for Andrology recommendations and the European Society of Human Reproduction and Embryology, semen analysis was done on all male participants [18]. The study included cases of infertility with ovulatory problems and male and unexplained diseases. The exclusion criteria included tubal and severe male disorders like oligospermia and genitourinary anatomic, ejaculation, and endocrine. Pregnancies were documented as a binary variable with 15 independent variables for each sample, nine of which were quantitative, and the rest were qualitative. Table 1 contains descriptive information for both pregnant and non-pregnant couples.

**Table 1:** Baseline parameters of the IUI candidates in both groups

Variable’s name	Category name	Success	Unsuccess	P value
Success rate^D^	–	155 (28)	391 (72)	
Female age (year)^T^	–	(24.98 ± 4.850)	(27.34 ± 6.588)	< 0.001
Male age (year)^T^	–	(29.85 ± 6.033)	(32.09 ± 8.037)	0.002
Duration of infertility (year)^T^	–	(2.91 ± 2.45)	(3.79 ± 3.15)	0.002
Cycle day of IUI (day)^T^	–	(15.3 ± 2.774)	(15.33 ± 6.033)	0.912
Sperm concentration^T^	–	(77.59 ± 25.8)	(79.58 ± 23.354)	0.386
Sperm motility (%)^T^	–	(61.94 ± 11.889)	(59.57 ± 9.444)	0.015
Sperm motility grading Score^T^	–	(1.9 ± 0.33)	(1.73 ± 0.22)	< 0.001
Number of Follicle on the day of HCG^M^	–	315	256	< 0.001
Number of previous IUI^M^	–	275	272	0.867
Type of infertility^C^	Primary	123 (29.3)	297 (70.7)	0.396
	Secondary	32 (25.4)	94 (74.6)	
Menstruation regularity^C^	Regular	83 (26.2)	234 (73.8)	0.179
	Irregular	72 (31.4)	157 (68.8)	
Galactorrhea^C^	Yes	20 (26.3)	56 (73.3)	0.666
	No	135 (28.7)	335 (71.3)	
Hirsutism^C^	Yes	58 (31.2)	128 (68.8)	0.298
	No	97 (26.9)	263 (73.1)	
Treatment with HMG^C^	Yes	1 (7.1)	13 (92.9)	0.074
	No	154 (28.9)	378 (71.1)	
Treatment with clomiphene-HMG^C^	Yes	73 (30.8)	164 (69.2)	0.273
	No	82 (26.5)	227 (73.5)	
Treatment with clomiphene^C^	Yes	81 (27.5)	214 (72.5)	0.601
	No	74 (29.5)	177 (70.5)	
Female factor^C^	Yes	53 (28.5)	133 (71.5)	0.968
	No	102 (28.3)	258 (71.7)	
Male factor^C^	Yes	34 (34)	66 (66)	0.168
	No	121 (27.1)	325 (72.9)	
Female and male pregnancy factors both^C^	Yes	10 (11.8)	75 (88.2)	< 0.001
	No	145 (31.5)	316 (68.5)	
Unexplained pregnancy factor^C^	Yes	58 (33.1)	117 (66.9)	0.091
	No	97 (26.1)	274 (73.9)	

Abbreviations of D, T, M, and C, respectively, are related by the dependent variable expressed as count (%), continuous variable expressed as mean (standard deviation), discrete variables expressed as mean rank, and categorical variables expressed as count (%). Also, abbreviations of T, M, and C, respectively related by The P values of the Independent T-test, Mann–Whitney test, and Chi-square tests

The minimum sample size required for this study, citing the article by NMF Buderer et al., and also considering the values of 25%, 5%, 70%, and 95% for prevalence, type 1 error, sensitivity, and specificity, respectively, was obtained equal to 440 samples [19].

### Parameter definition

All parameters were taken from the patient's records. Some of them, such as galactorrhea or hirsutism, were reported according to physical examination. All patients were treated with Human Menstrual Gonadotropin (HMG), Clomiphene Citrate, or a combination. The cycle day of IUI was measured from the first day of menstruation (bleeding, not spotting) to 36 h after Human Chorionic Gonadotropin administration. Clinical pregnancy in IUI was considered a gestational sac in the uterus, confirmed by transvaginal sonography. Sperm motility was classified into three grades; (a) progressive with three scores, (b) moderate with two scores, and (c) poor and immotile with one score [20].

Infertility duration was calculated by failing to achieve a clinical pregnancy after 12 months or more of regular unprotected sexual intercourse [21]. The number of follicles on the day of HCG was measured on transvaginal sonography. Primary infertility was defined as infertility of a woman who has never been pregnant and secondary infertility is the infertility of a woman with at least one history of pregnancy before [22]. Unexplained pregnancy was defined as the couple's inability to conceive without any identifiable factor (ovulatory cycles, patent tube, and normal semen analysis) [23].

### Statistical analysis

In this work, univariate analysis was performed following an initial preparation of the data to handle outliers and missing data. The qualitative factors were then classified as dummy variables, and the data was normalized before being utilized to construct the predictive model.

Choosing the smallest and most relevant set of variables may make impressive affect in the accuracy and speed of the analysis results. As a consequence, we used feature selection approaches such as filter [24], wrapper [25], and embedded-based [26] to show the impact of the optimal feature set on the predictive capabilities of the models. Mutual Information Classification feature selection (MIC-FS), genetic algorithm feature selection (GA-FS), and random forest feature selection (RF-FS) [26] are the algorithms utilized in these approaches. Moreover, to demonstrate the differences caused by feature selection, we fit all of the models once without feature selection (W-FS).

### Imbalance data

In many cases, experts seek to detect abnormalities, such as fraud detection, intrusion detecting, rare medical cases detection, etc. In anomaly detection, the goal is to find cases that differ from most patients. The data relating to the results of IUI methods often have an imbalance due to the relatively low success rate. In this study, 28% (155) cases of the data related to the success while 72% (391) cases were of the IUI failure, so the imbalance ratio for this study was equal to 39% (391/155). The previous studies showed that the performance of statistical models fitted with balanced data showed better results than the imbalanced data. When statistical models are fitted with imbalanced data, they are often biased towards the majority class and show poor performance in predicting the minority class [27–29]. Nevertheless, it is impossible to say how much imbalance in the distribution of the classes affects classification performance because other elements like sample size, relevance of predictor variables, etc. all have an impact the model's effectiveness. As a result, different fields have varied levels of effectiveness when using imbalanced data in predicting models [30].

### Resampling methods

The classification of imbalanced data is one of the most complex issues in machine learning. When data classes are significantly imbalanced, the performance of a classification learning system is substantially degraded. In this scenario, we may have excellent overall accuracy, but this accuracy is affected by the majority class’s higher weight. On the other hand, it performs poorly in predicting the minority class. This issue becomes much more concerning when the researcher prioritizes minority class prediction.

One strategy for solving this problem is to use resampling methods to balance the distribution of classes by adding or removing data samples. Oversampling and undersampling are the two approaches used in resampling. Two techniques, Smote-Tomek (Stomek) and Smote-ENN(SENN), the combination of methodologies described above, were utilized in this investigation [31, 32].

### Model fitting

Five machine learning models were used to predict the success of IUI, including logistic regression (LR), Support Vector Classification (SVC), random forest (RF), Extreme Gradient Boosting (XGBoost) and Stacking generalization (Stack), which were a combination of four first classifiers [13, 33–37]. More details regarding the models are described in Additional file 1 (see Sect. 1 in Additional file 1).

### Model evaluation

**Fig. 1:**
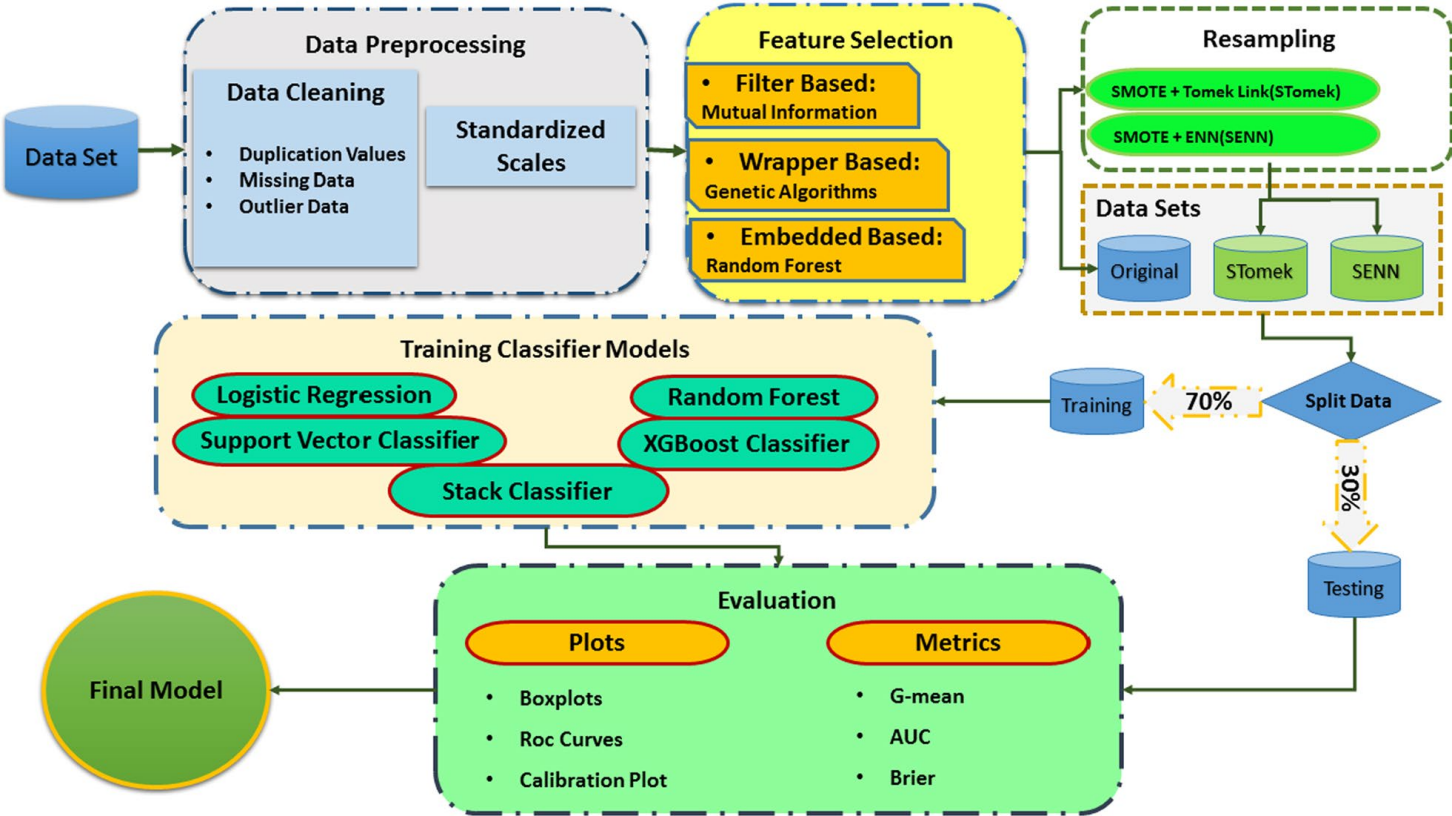
Modeling steps with Python in this study

We utilized three distinct-based techniques for model assessment and comparison: Boxplot [38], ROC curve, and calibration plot. A practical tool to show the caliber of predictions of a classification model, a classifier model has well-calibrated predicted probabilities when actual observed cases occur that coincident the predicted cases [39] with the Geometric mean(Gmean) [40, 41], Area under the curve (AUC) [39], brier score. The evaluation measure that causes discrimination and calibration at the same time [42] and, Delong test [43] for model evaluation and comparison. The details related to each mentioned criteria are explained in the second part of the Additional file 1 (see Additional file 1). Figure 1 shows a flowchart of our research modeling procedures.

## Results

### Descriptive

According to the results of this study, 155 (28%) couples treated with IUI experienced pregnancy success. Among couples who had a successful pregnancy, the range of age (the average) of women and men was 16–42 (24.98) and 21–72 (29.85) years old, respectively. The duration of infertility and cycle day of IUI for these people was 2.9 years and 15.3 days on average.

To test the relationship between the main demographic and independent variables and the response variable, we used the Chi-square test for qualitative variables and t-test and Mann–Whitney test for continuous and discrete variables. A p-value < 0.05 was considered statistically significant. The results are shown in Table 1.

According to this table, 7 out of the 15 variables had a significant relationship with fertility success: female age (p < 0.001), male age (p = 0.002), duration of infertility (p = 0.002), sperm motility (p = 0.015), sperm motility grading score (p < 0.001), number of Follicle on the day of HCG (p < 0.001) and female and male pregnancy factors both (p < 0.001).

### Feature selection

The MIC-FS method, ten variables, GA-FS method 14 variables, and RF-FS method eight variables out of 21 were selected as the best variables set for the model. An overview of the steps for determining the optimal feature set for all three methods is shown in Sect. 3 in Additional file 1 (see Additional file 1).

Female and male age, sperm concentration, sperm motility, duration of infertility, sperm motility grading score, number of follicles on the day of HCG, and female and male pregnancy factors were selected as the best variables in the RF-FS method. It is noticeable that the GA-FS selected all features of the above list, and the first 6 of those listed were among the features chosen by the MIC-FS method.

### Model development

To achieve the optimal performance in each model, we used the Grid-Search command from the Sklearn package with five folds of random duplicates of data (see selected optimal parameters in Sect. 4 of Additional file 1).

The optimal values for the parameters of the classification models were selected [44], and using Stomek, SENN resampling methods, two balanced datasets of the original data were created(via the Imblearn package in Python). Then, each data was divided by a ratio of 70% for training and 30% for testing. The models were trained and tested with each selected feature set and for all features for each data.

### Model validation

In this study, we used three different methods to evaluate and compare the models which are as follows.

**Fig. 2:**
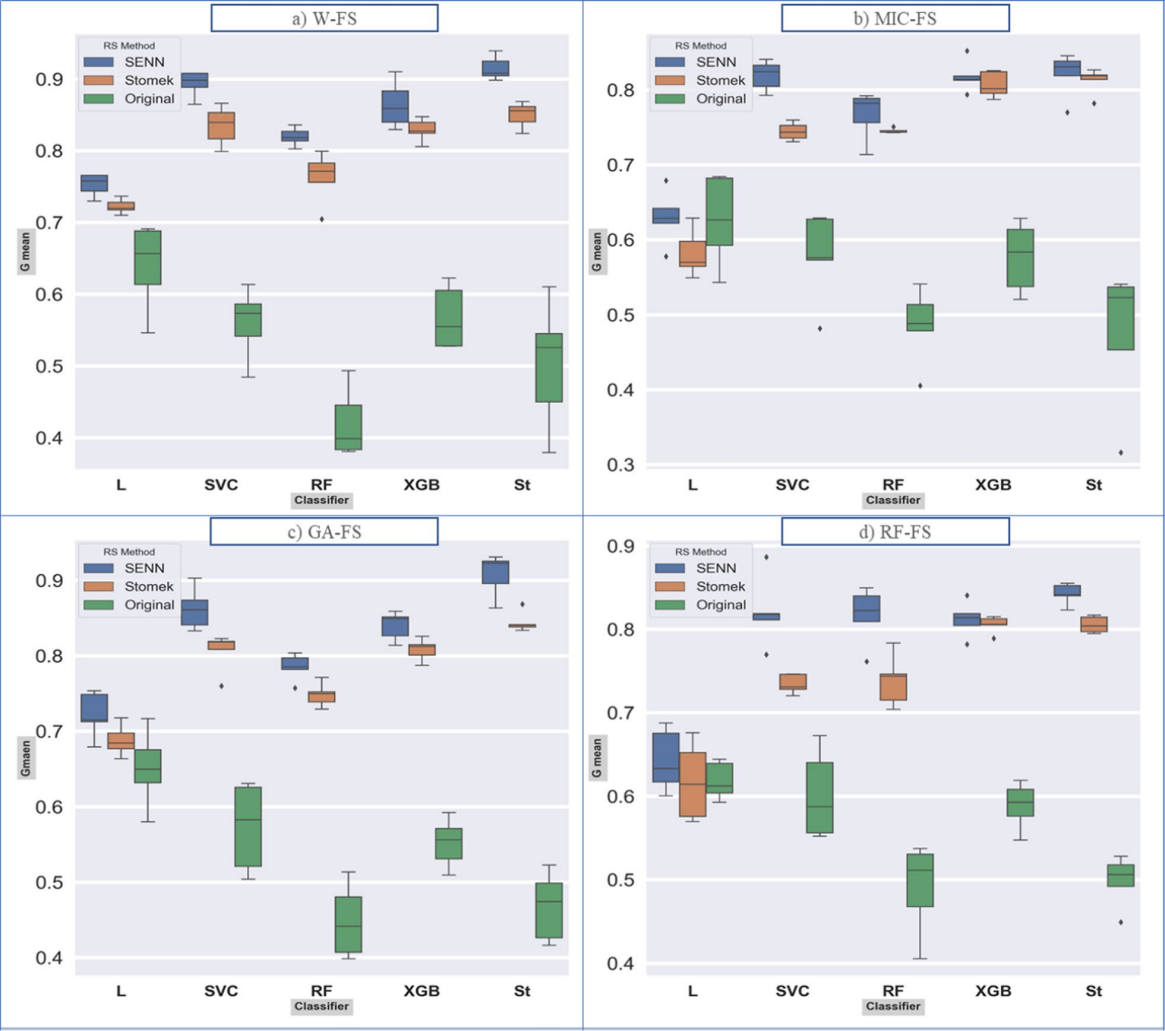
Boxplot for G-means index, for each model. a: d show plots related to the feature selection methods. Abbreviations: RS method: Resampling method

**Fig. 3:**
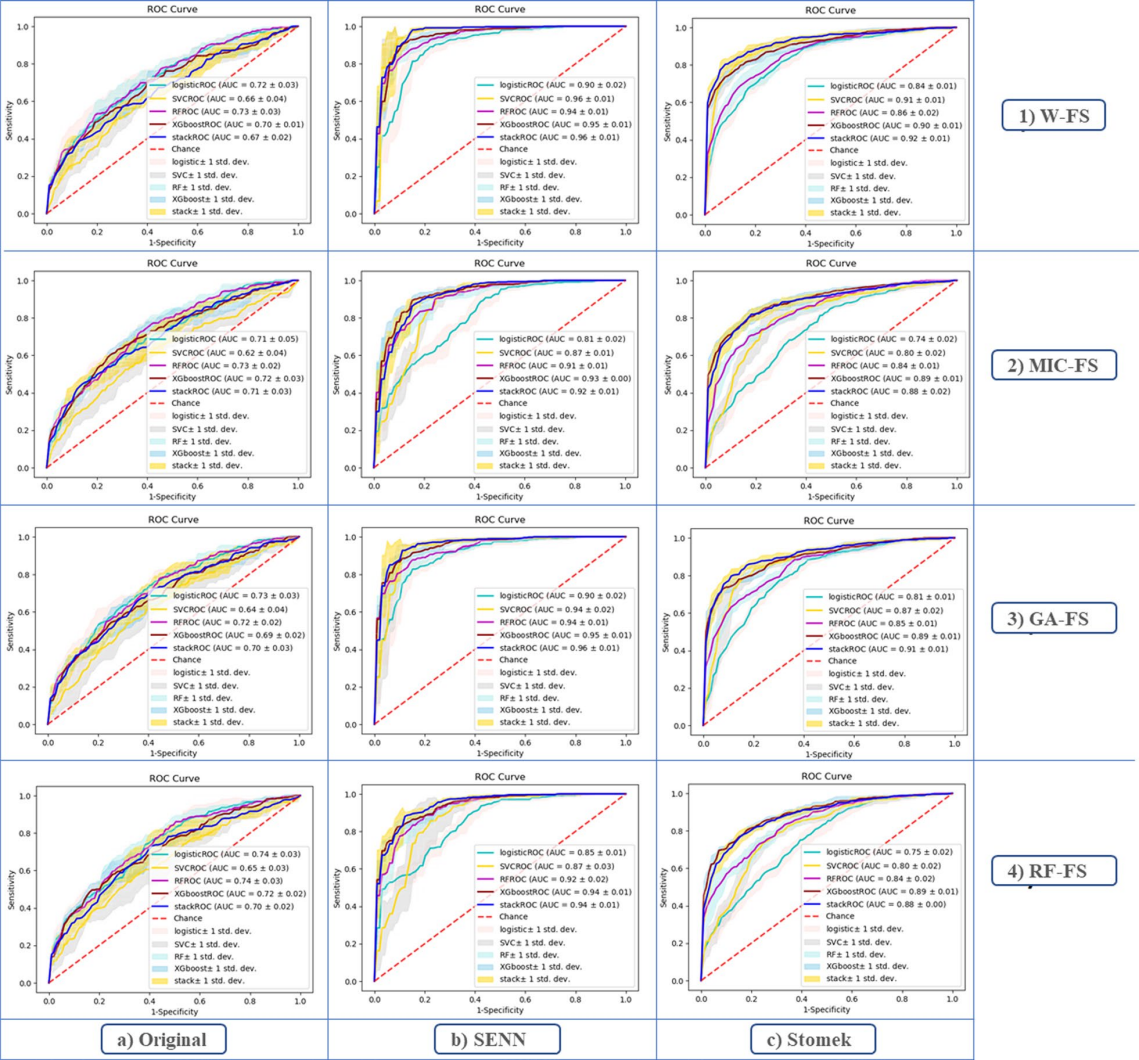
ROC curve and AUC index of each class by different models. Each row by 1: 4 numbers show graphs for each feature selection method and Columns a: c show plots related to the data used to model training

*Boxplot and ROC curve* Separately for models (Fig. 2) and find that the changes of the fit models with balanced datasets were less than the original dataset. Also, according to Fig. 3, For the SENN dataset, the maximum and minimum AUC values were 96% and 81% for the Stack model, equally for the W_FS and GA_FS techniques, and the LR model in the MIC-FS technique, respectively. (AUC and Gmean averages for all models are 82.1% and 80.7%, respectively). For the Stomek dataset, the maximum and minimum AUC values were 92% and 74% for the Stack model in the W_FS technique and the LR model in the MIC-FS technique, respectively. (AUC and Gmean averages for all models are 77.3% and 76.4%, respectively, in this dataset). Finally, for the original dataset, the maximum and minimum AUC values were 74% and 62% achieved equally for the Stack and LR models in RF_FS techniques and the SVC model in the MIC-FS technique, respectively. (AUC and Gmean averages for all models are 61.4% and 54.6%, respectively, in this dataset). Besides, the average AUC and Gmean for the fitted models according to the feature selection techniques in descending order is equal to 75.7% and 72.9%, 74.4% and 71.4%, 72.5% and 69.6%, 71.8%, and 68.6% respectively for the W-FS, GA-FS, RF-FS, and MIC-FS techniques. Furthermore, the superiority of the two models, Stack and XGBoost, is evident in this plot.

**Fig. 4:**
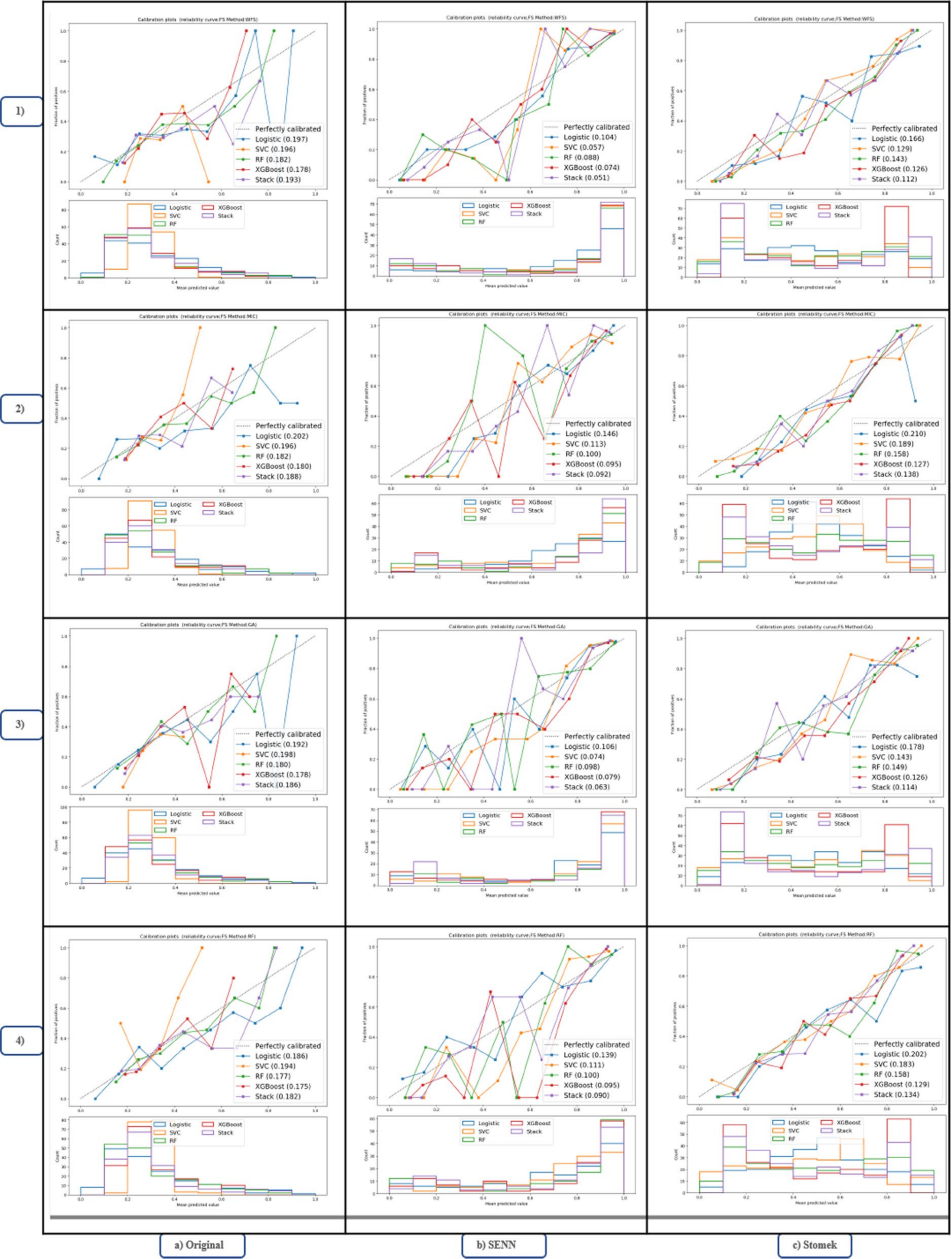
Reliability and predictive power of each class by different model. Each row by 1: 4 numbers show graphs for each feature selection method; 1) Without feature selection (W_FS), 2) Mutual Information Classification feature selection (MIC-FS), 3) genetic algorithm feature selection (GA-FS), and 4) random forest feature selection (RF-FS), and Columns a: c show plots related to the data used to model training

*Calibration plot* In Fig. 4, each graph consists of two parts. The first part shows the reliability, and the second shows each class's predictive power using different models.

According to the first part of this graph and its comparison of the three datasets, the models fitted with Stomek data are more well-calibrated than those with the other two datasets. Moreover, in this data set, models fitted with the RF-FS technique are more well-calibrated than models fitted with different techniques. On the other hand, models fitted with SENN data show poor calibration for all feature selection methods.

In reviewing the second part of Fig. 4 for different datasets, models fitted with the original data are more focused on predicting the negative class (IUI failure class). In contrast, models fitted with the SENN data are more focused on predicting the positive class (IUI success class). Unlike the previous two datasets, the models fitted with Stomek data maintain equilibrium in predicting both classes.

### Model selection

**Fig. 5:**
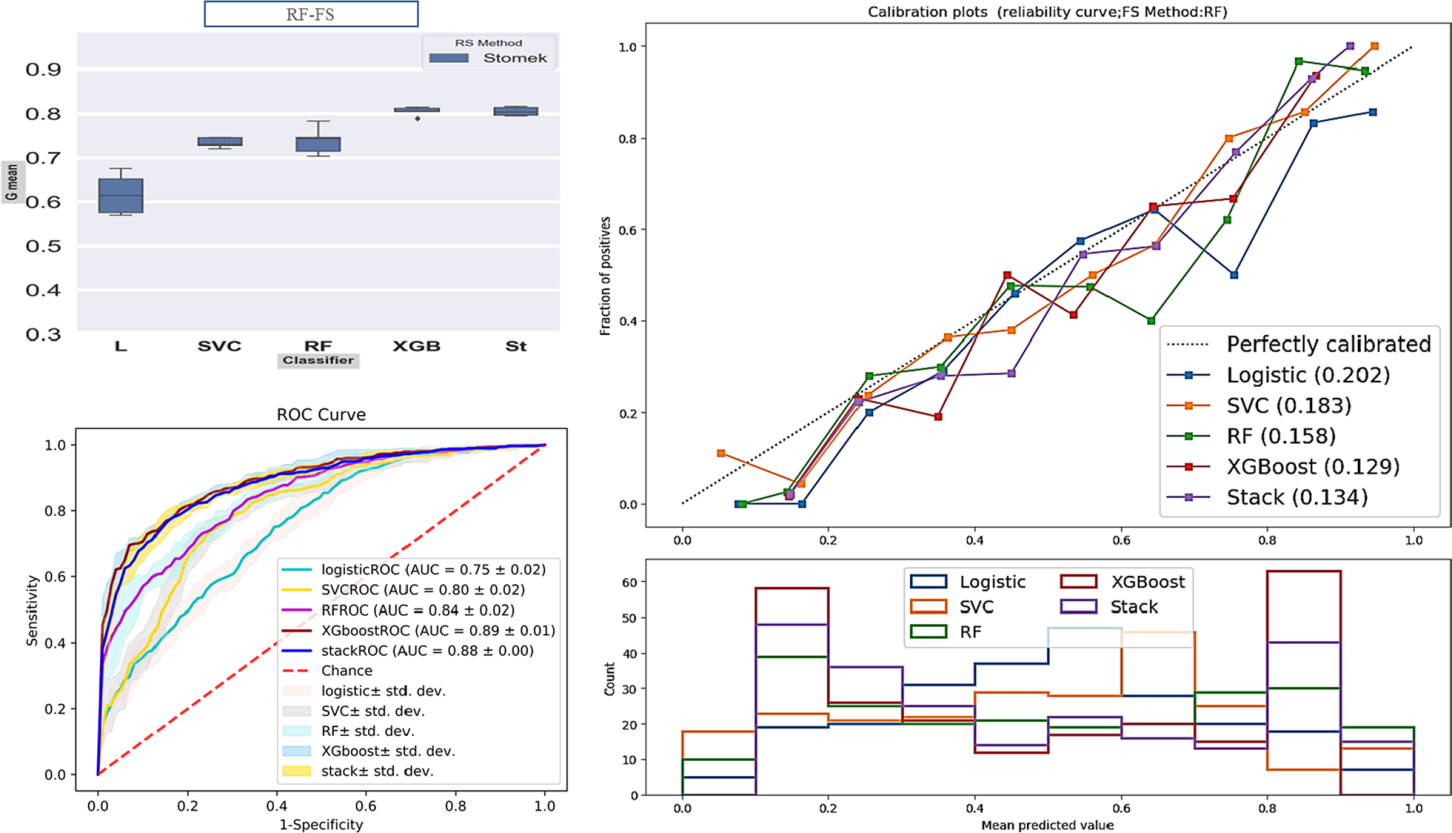
Boxplot, calibration plot, and ROC curve for trained models with random forest- selected features from the Stomek-balanced dataset

After reviews, the Stomek resampling method was selected as the best resampling method. The RF-FS technique was chosen as the best feature selection method; ultimately, the XGBoost and Stack models achieved the best grading performance against the models. However, they had no statistically significant difference (Delong p-value > 0.05). Still, for reasons mentioned in the discussion, the XGBoost model was chosen as the best model in this study. Table 2 shows the evaluation values, and Fig. 5 Including the ROC curve, Calibration, and Boxplots of the trained models by optimal features selected from RF-FS.

**Table 2:** Performance values for trained models by RF-FS from the Stomek-balanced dataset

Classifier	AUC	Brier	G mean
LR	0.75	0.202	0.618
SVC	0.80	0.183	0.734
RF	0.84	0.158	0.739
XGBoost	0.89	0.129	0.806
Stack	0.88	0.134	0.805

**Fig. 6:**
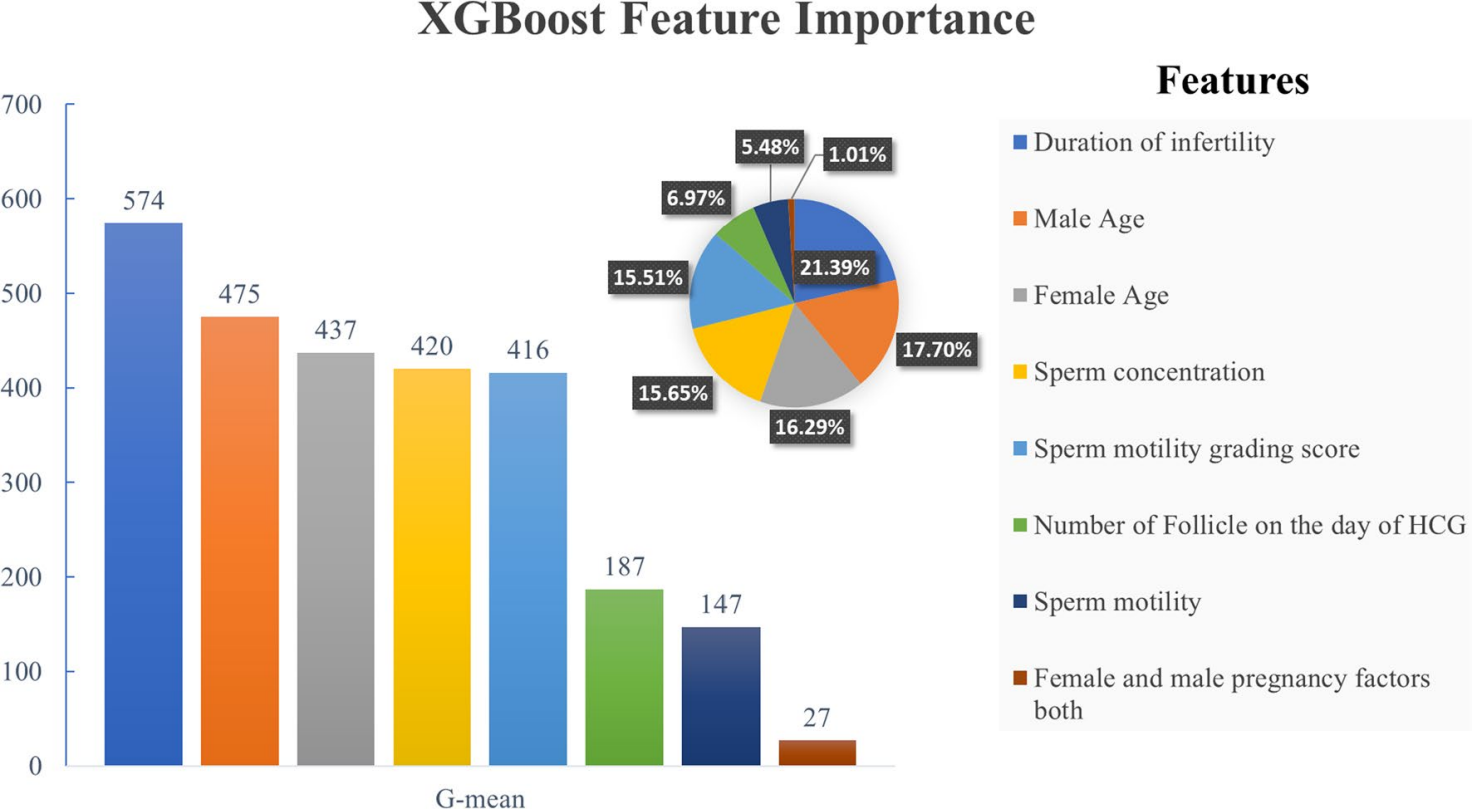
Ranking of features used in XGBoost based on the effect on model learning and prediction

Figure 6 ranked the features used in XGBoost based on the effect on learning and model prediction that duration of infertility, male and female age, sperm concentration, and sperm motility grading score were the most practical features in improving the prognosis.

## Discussion

According to previous studies' statistics, this approach's success has been between 18 and 30% [45]. In this study, 28% of individuals undergoing IUI treatment obtained a successful pregnancy. Among couples with successful pregnancy, the average age of women and men was 24.98 and 29.85 years, respectively. The duration of infertility and cycle day of IUI for these people was 2.9 years and 15.3 days on average. Since this method cannot guarantee pregnancy and may even lead to complications such as OHSS, evidence-based tools for the probability of successful live birth before IUI treatment are needed to aid in patient counseling in clinical practice.

In this study, XGBoost, by achieving Gmean, AUC, Brier values of 0.80, 0.89, and 0.129, respectively, presented the best performance compared to other learning algorithms and the most predictable factor in IUI success was infertility duration of couples. Given the high rate of infertility among couples worldwide, the importance of IUI methods is quite palpable. Various methods have been developed in this field, but none of these methods can guarantee pregnancy success to patients. Since these treatments are costly and time-consuming, the need for accurate methods of predicting the success of these methods is felt more than ever because, in this case, the patient will accept or reject the treatment with more awareness of the possibility of successful treatment. Besides, the doctor can prescribe the appropriate treatment for the patient faster and with less time.

Machine learning models have been developed in various fields, including data classification, which is highly practical and attractive in the real world. As we know, the medical world is full of binary data suitable for fitting classifier models. A study by Cline Blank et al. was performed to predict pregnancy success in IVF. Random forest and logistic regression methods were used for prediction, and the AUC performance index was 0.84 and 0.66%, respectively, which showed the superiority of the random forest model [7]. Although various tools have been used for evaluation in their study, it seems that the place of the calibration plot tool to improve assessment in this group is empty.

Another study was conducted by Jiahui Qu et al. to predict live births in IVF using machine learning algorithms with logistic, random forest, XGBoost, and SVM prediction models. Finally, the XGBoost algorithm with 0.70 and 0.73 values for accuracy and AUC was introduced as the best model [46]. In a study conducted by Md Rafiul Hassan et al. to inquiring about machine learning methods for predicting pregnancy using IVF, twelve studies on this subject were reviewed by this team, and only six studies used feature selection methods in their research. Moreover, in their study, different algorithms were trained by a selected set of features by the hill-climbing wrapper method [47]. Another study by Robert Milewski et al. classified IVSI ICSI/ET data using SVM and random forest algorithms. Finally, the same algorithm with 79% accuracy was recognized as the top model of this study [48].

A certain point in all the above studies lacks reference to the class imbalance in the data in this field. Additionally, except for Md Rafiul Hassan et al.'s analysis, the rest did not use any different feature selection method to select the optimal feature set. This study utilizes the machine learning classification models to classify the success rate of intrauterine insemination treatment methods showing high performance. According to this model, the duration of infertility was the most crucial factor in the success of IUI, and in the second was the ages of males and females. Hence, early initiation of infertility work-up seems wise and improves pregnancy.

In this work, we compared the performance of the models with different measures plus employed various feature selection and data balancing methods to fit the models. This study did not use conventional tools such as the ROC curve, AUC, and accuracy criterion as the main measures for comparing models because these measures are limited when the data are class imbalanced [49, 50]. In this circumstance, these indicators must employ the optimal point for a fair assessment [39]. The majority of the studies described above, as well as many others, have employed these criteria despite this problem, which can result in an unrealistic evaluation. The Gmean index is a method used to find the optimal point for the mentioned criteria, while this index is a criterion that can work well in unbalanced data [41]. Finally, we used the calibration plot to ensure our final model that can provide more confidence in the well-calibrated predictions of IUI success.

Feature selection can improve the quality of model learning by choosing the top features, eliminating ineffective features in learning, and ultimately improving model prediction. As mentioned, we used three feature selection methods with different bases (Filter-based, Wrapper-based, Embedded-based) to cover this weakness. After reviewing these three methods, according to Fig. 2, MIC-FS trained models show the highest difference, and GA-FS trained models to show the slightest difference compared to the W-FS trained models. Concerning Fig. 3, the GA-FS method has the smallest difference compared to trained models with all features. Up to this point, the GA-FS feature selection method seemed to work best. Still, by checking out the calibration plot, we found that the RF-FS method, although weaker in prediction than the GA-FS, had well-calibrated prediction for all models compared to other feature selection methods. Likewise, it is less complicated than other methods; this method has predicted the model with eight variables, while the MIC-FS method with ten and the GA-FS method with 14 variables have been trained and attempted to predict.

On the other hand, since the success rate for the IUI method is relatively low, the available data may have imbalance classes. Moreover, this leads to a learning bias in learning-based models towards the majority class [51]. In this study, we used two different methods, SENN and Stomek, to match data classes to solve this problem. A clear advantage of the SENN method was seen in the Boxplot and ROC curve to evaluate the best resampling method. However, according to the calibration plot, this method shows high predictive power and sensitivity for all models. On the other side, poor calibration is evident in all models trained with this dataset than in the other two datasets. This factor led to the abandonment of this method in favor of the Stomek resampling method since the models trained by the original data had insufficient predictive power for the positive class and had poor calibration than the balanced dataset by the Stomek method.

Meanwhile, the models trained with data balanced by the Stomek method, in addition to increasing the model’s predictive power for the positive class (IUI success), also increased the calibration of the models compared to the other two datasets. Therefore, to select the final model, we examined the trained models more closely with the Stomek method's resampled data and the features chosen by the RF-FS method. According to Figs. 2, 3 and 4, it is clear that the two XGBoost and Stack models perform better than the other models and especially the multiple logistic regression model, which is known as the traditional and standard model. Although there was no significant difference between the two models, the Brier score for this model (0.129) is lower than the Stack model (0.134), and the XGBoost model is also less complicated in terms of complexity, indicating its superiority.

Despite the strengths of this research, not using multicenter data and the lack of external validity is the limitations of our study, which were not possible due to time and financial constraints.

## Conclusion

In this study, we tried to develop and present appropriate models based on machine learning to predict IUI methods' success, identify problems related to the data obtained in this field, and provide ways to cover them. It is expected that by gathering valuable and exclusive features in this field, to train the mentioned models, especially model XGBoost, it will be possible to achieve powerful predictive models in the future to help specialists in IUI success prediction. As a result, the assurance of the experts in this field will seek the correctness of the counseling for the referring couples regarding the possibility of IUI success for them, uniquely through the specific characteristics of each couple. Also, increasing the probability of a successful pregnancy, reducing costs, and avoiding wasted time can be the indirect result of a consultation with a high chance of choosing the appropriate treatment method for patients in this department.

## Supplementary Information

**Additional file 1. Section 1:** Details of classifier models of study. **Section 2:** Definition of used Evaluation measures. **Section3:** An overview of the steps for selecting the optimal feature set forall three methods. **Section 4:** Optimal parameters.

## Data Availability

The datasets used and/or analyzed during the current study available from the corresponding author on reasonable request.

## References

[1] Pan MM, Hockenberry MS, Kirby EW, Lipshultz LI (2018). Male infertility diagnosis and treatment in the era of in vitro fertilization and intracytoplasmic sperm injection. Med Clin.

[2] Muthigi A, Jahandideh S, Bishop LA, Naeemi FK, Shipley SK, O’Brien JE, Shin PR, Devine K, Tanrikut C (2021). Clarifying the relationship between total motile sperm counts and intrauterine insemination pregnancy rates. Fertil Steril.

[3] Merviel P, Labarre M, James P, Bouée S, Chabaud J-J, Roche S, Cabry R, Scheffler F, Lourdel E, Benkhalifa M (2022). Should intrauterine inseminations still be proposed in cases of unexplained infertility? Retrospective study and literature review. Arch Gynecol Obstet.

[4] Nesbit CB, Blanchette-Porter M, Esfandiari N (2022). Ovulation induction and intrauterine insemination in women of advanced reproductive age: a systematic review of the literature. J Assist Reprod Genet.

[5] Guzick DS, Carson SA, Coutifaris C, Overstreet JW, Factor-Litvak P, Steinkampf MP, Hill JA, Mastroianni L, Buster JE, Nakajima ST (1999). Efficacy of superovulation and intrauterine insemination in the treatment of infertility. N Engl J Med.

[6] T Kundnani M, Dalal R, Palshetkar NP, D Pai H: Complications of intrauterine insemination.

[7] Blank C, Wildeboer RR, DeCroo I, Tilleman K, Weyers B, De Sutter P, Mischi M, Schoot BC (2019). Prediction of implantation after blastocyst transfer in in vitro fertilization: a machine-learning perspective. Fertil Steril.

[8] Zarinara A, Zeraati H, Kamali K, Mohammad K, Shahnazari P, Akhondi MM (2016). Models predicting success of infertility treatment: a systematic review. J Reprod Infertil.

[9] Leushuis E, Van der Steeg JW, Steures P, Bossuyt PMM, Eijkemans MJC, Van der Veen F, Mol BWJ, Hompes PGA (2009). Prediction models in reproductive medicine: a critical appraisal. Hum Reprod Update.

[10] Sedehi M, Mehrabi Y, Kazemnejad A, Hadaegh F (2010). Comparison of artificial neural network, logistic regression and discriminant analysis methods in prediction of metabolic syndrome. Iran J Endocrinol Metab.

[11] Milewski R, Milewska AJ, Więsak T, Morgan A (2013). Comparison of artificial neural networks and logistic regression analysis in pregnancy prediction using the in vitro fertilization treatment. Stud Logic Gramm Rhetor.

[12] Sidey-Gibbons JAM, Sidey-Gibbons CJ (2019). Machine learning in medicine: a practical introduction. BMC Med Res Methodol.

[13] Kotsiantis SB, Zaharakis ID, Pintelas PE (2006). Machine learning: a review of classification and combining techniques. Artif Intell Rev.

[14] Wasserman L. The role of assumptions in machine learning and statistics: dont drink the koolaid. In*.*: Technical report, Carnegie Mellon University; 2015. p. 8.

[15] Singh A, Thakur N, Sharma A. A review of supervised machine learning algorithms. In: IEEE; 2016. pp. 1310–5.

[16] Ombelet W, Dhont N, Thijssen A, Bosmans E, Kruger T (2014). Semen quality and prediction of IUI success in male subfertility: a systematic review. Reprod Biomed Online.

[17] Allahbadia GN (2017). Intrauterine insemination: fundamentals revisited. J Obstetr Gynecol India.

[18] Kvist U, Giwercman A, Haugen TB, Suominen J, Bjorndahl L. Manual on basic semen analysis NAFAESHRE 4th edn. Cambridge; 2001. p. 1–32.

[19] Buderer NMF (1996). Statistical methodology: I. Incorporating the prevalence of disease into the sample size calculation for sensitivity and specificity. Acad Emerg Med.

[20] Cooper TG, Noonan E, Von Eckardstein S, Auger J, Baker HW, Behre HM, Haugen TB, Kruger T, Wang C, Mbizvo MT (2010). World Health Organization reference values for human semen characteristics. Hum Reprod Update.

[21] Zegers-Hochschild F, Adamson GD, De Mouzon J, Ishihara O, Mansour R, Nygren K, Sullivan E, Van der Poel S (2009). The international committee for monitoring assisted reproductive technology (ICMART) and the world health organization (WHO) revised glossary on ART terminology, 2009. Hum Reprod.

[22] Tabong PT-N, Adongo PB (2013). Infertility and childlessness: a qualitative study of the experiences of infertile couples in Northern Ghana. BMC Pregnan Childb.

[23] Nardo LG, Chouliaras S. Definitions and epidemiology of unexplained female infertility. In: Unexplained infertility. Springer; 2015. p 21–5.

[24] Tang J, Alelyani S, Liu H. Feature selection for classification: a review. Data classification: algorithms and applications; 2014. p. 37.

[25] Masoudi-Sobhanzadeh Y, Motieghader H, Masoudi-Nejad A (2019). FeatureSelect: a software for feature selection based on machine learning approaches. BMC Bioinform.

[26] Paja W. Generational feature selection using random forest approach. In: IEEE; 2019. p 354–7.

[27] Le T, Hoang Son L, Vo MT, Lee MY, Baik SW (2018). A cluster-based boosting algorithm for bankruptcy prediction in a highly imbalanced dataset. Symmetry.

[28] Abd Elrahman SM, Abraham A (2013). A review of class imbalance problem. J Netw Innov Comput.

[29] Liu C, Wu J, Mirador L, Song Y, Hou W. Classifying dna methylation imbalance data in cancer risk prediction using smote and tomek link methods. In: Springer; 2018. P. 1–9.

[30] Sun Y, Wong AKC, Kamel MS (2009). Classification of imbalanced data: a review. Int J Pattern Recognit Artif Intell.

[31] Wang ZHE, Wu C, Zheng K, Niu X, Wang X (2019). SMOTETomek-based resampling for personality recognition. IEEE Access.

[32] Batista GE, Prati RC, Monard MC (2004). A study of the behavior of several methods for balancing machine learning training data. ACM SIGKDD Explor Newsl.

[33] Bhavsar H, Ganatra A (2012). A comparative study of training algorithms for supervised machine learning. Int J Soft Comput Eng.

[34] Lai K, Twine N (2018). O’brien A, Guo Y, Bauer D: Artificial intelligence and machine learning in bioinformatics. Encycl Bioinform Comput Biol ABC f Bioinform.

[35] Mushtaq MS, Mellouk A. Quality of experience paradigm in multimedia services: application to OTT video streaming and VoIP services. Elsevier; 2017.

[36] Torlay L, Perrone-Bertolotti M, Thomas E, Baciu M (2017). Machine learning–XGBoost analysis of language networks to classify patients with epilepsy. Brain Inform.

[37] Sesmero MP, Ledezma AI, Sanchis A (2015). Generating ensembles of heterogeneous classifiers using stacked generalization. Wiley Interdiscip Rev Data Min Knowl Discov.

[38] Spitzer M, Wildenhain J, Rappsilber J, Tyers M (2014). BoxPlotR: a web tool for generation of box plots. Nat Methods.

[39] Vuk M, Curk T (2006). ROC curve, lift chart and calibration plot. Adv Methodol Stat.

[40] Akosa J. Predictive accuracy: a misleading performance measure for highly imbalanced data. In: 2017. p 1–4.

[41] Mahin M, Islam MJ, Debnath BC, Khatun A. Tuning distance metrics and k to find sub-categories of minority class from imbalance data using k nearest neighbours. In: IEEE; 2019. p. 1–6.

[42] Blattenberger G, Lad F (1985). Separating the Brier score into calibration and refinement components: a graphical exposition. Am Stat.

[43] DeLong ER, DeLong DM, Clarke-Pearson DL (1988). Comparing the areas under two or more correlated receiver operating characteristic curves: a nonparametric approach. Biometrics.

[44] Buitinck L, Louppe G, Blondel M, Pedregosa F, Mueller A, Grisel O, Niculae V, Prettenhofer P, Gramfort A, Grobler J. API design for machine learning software: experiences from the scikit-learn project. arXiv preprint arXiv:13090238 2013.

[45] Madhuri MS, Thyagaraju C, Naidu A, Dasari P (2022). The effect of endometrial scratching on pregnancy rate after failed intrauterine insemination: a randomised controlled trail. Eur J Obstet Gynecol Reprod Biol.

[46] Qiu J, Li P, Dong M, Xin X, Tan J (2019). Personalized prediction of live birth prior to the first in vitro fertilization treatment: a machine learning method. J Transl Med.

[47] Hassan MR, Al-Insaif S, Hossain MI, Kamruzzaman J (2020). A machine learning approach for prediction of pregnancy outcome following IVF treatment. Neural Comput Appl.

[48] Milewski R, Malinowski P, Milewska AJ, Ziniewicz P, Czerniecki J, Pierzyński P, Wołczyński S (2012). Classification issue in the IVF ICSI/ET data analysis. Stud Logic Gramm Rhetor Log Stat Comput Methods Med.

[49] García V, Sánchez JS, Mollineda RA (2012). On the effectiveness of preprocessing methods when dealing with different levels of class imbalance. Knowl Based Syst.

[50] Yang S, Berdine G (2017). The receiver operating characteristic (ROC) curve. Southw Respirat Crit Care Chronicl.

[51] Kaur H, Pannu HS, Malhi AK (2019). A systematic review on imbalanced data challenges in machine learning: applications and solutions. ACM Comput Surv.

