# Supplementary material for: Developing machine learning-based models to predict intrauterine insemination (IUI) success by address modeling challenges in imbalanced data and providing modification solutions for them
Source: BMC Med Inform Decis Mak. 2022 Sep 1;22:228. doi: 10.1186/s12911-022-01974-8 (PMC9434923; doi:10.1186/s12911-022-01974-8)
Supplement: Supplementary file 1 — Additional file 1. Section 1: Details of classifier models of study. Section 2: Definition of used Evaluation measures. Section3: An overview of the steps for selecting the optimal feature set forall three methods. Section 4: Optimal parameters. [file 12911_2022_1974_MOESM1_ESM.docx]

**Contents**

1. **Section 1: Details of classifier models of study**
2. **Section 2: Definition of used Evaluation measures**
3. **Section 3: An overview of the steps for selecting the optimal feature set for all three methods**
4. **Section 4: Optimal parameters**

**Section 1: Details of classifier models of study:**

Five supervised classification learning models were trained using Python V 3.7 programming language, which are as follows:

**Logistic regression (LR):** We can use logistic regression when our dependent variable is two-dimensional (such as gender, disease, or non-disease), and we want to make predictions through logical functions. Logistic regression has the following form:

$$log[ \frac{\pi}{1-\pi}]=\alpha+\sum_{i}^{k} \beta_{i}.X_{i}$$

In this model, π is the probability that a person belongs to the first level of the dependent variable, $X_{i}$ and $\beta_{i}$ are the independent variable and the model coefficient for the independent variable $i$, respectively.

**Support Vector Machine:** One of the most popular and influential supervised learning models is that used for classification (SVC) and regression (SVR). This model uses the construction of pages in multidimensional space to separate classes into categories. One of the best advantages of SVC is the effectiveness of this model in multidimensional spaces and even when the number of features is more than the samples. The linear state equation for this model is as follows:

$$y_{i}=Sgn\left( w^{T}x+b \right)$$

This equation uses linear kernel function ($K\left( \mathcal{X}_{\mathfrak{i}}, \mathcal{X}_{\mathfrak{j}} \right)=\mathcal{X}_{\mathfrak{i}}. \mathcal{X}_{\mathfrak{j}}$) for linearly separable data. Based on the methods of selecting the optimal parameters for this model, the Radial Basis Function Kernels (RBF) (($K\left( \mathcal{X}_{\mathfrak{i}}, \mathcal{X}_{\mathfrak{j}} \right)={exp\mathcal{(||X}}_{\mathfrak{i}}- \mathcal{X}_{\mathfrak{j}}||^{2}/\sigma^{2}$) is used.

**Random Forest:** Random Forest (RF) is an assembly classification consisting of several decision-tree models (DTs) for higher predictive performance. RF trains each tree with training data by creating classification trees and using a bootstrap sample technique. Finally, it performs the classification operation with the majority vote of the trees in each class. Equation 1 shows the Decision function for the model:

$$H\left( x \right)=arg\max_{y} \sum_{i=1}^{k} I(h_{i}\left( x \right)=Y)$$

Where x, $h_{i}$, Y, I, and H are test sample, single Decision Tree, output variable(labels), indicator function, and Random Forest model, respectively.

**Extreme Gradient Boosting (XGBoost):** This is a learning assembly model for classification and regression based on the Gradient Boosting Decision Tree (GBDT) and is widely used in data mining. Some of this model's essential advantages are High scalability, parallelism, and fast execution. For data set D = [(${(x}_{\mathfrak{i}},y_{\mathfrak{i}}) (x_{\mathfrak{i}} \in R^{m}, y_{\mathfrak{i}} \in R, \mathfrak{i}=1, 2 , \ldots, n$)] containing n*m dimensions, the XGBoost model can be expressed as $\hat{y}_{i}= \sum_{k=1}^{k} f_{k}\left( x_{i} \right), f_{k} \in F(i=1, 2, \ldots, n)$ in which is $F=\left[ f\left( x \right)= W_{q\left( x \right)} \right](q: R^{m} \to\left\{ 1, 2, \ldots, T \right\}, W \in R^{T})$ is the classification decision tree structure set, q in the tree structure of the sample map to leaf nodes, T is the number of leaf nodes, and W is the actual score of the leaf nodes.

**Stacking generalization (Stack):** Unlike other assembly algorithms such as Bagging and Boosting, which create an assembly classification of homogeneous ensembles, The Stack algorithm makes an assembly algorithm of heterogeneous classifiers. Hence, each algorithm has a different way of presenting knowledge and a different training base. The hypothesis of this model uses different perspectives to generate a consensus of different classifications; therefore, it is expected that the prediction composition of those algorithms will be more accurate than the individual prediction of each model.

In this study, we used this algorithm to combine the four algorithms we mentioned.

**Section 2: Definition of used Evaluation measures:**

It is practically useless to use the Accuracy index to evaluate classifiers on unbalanced data because the high value of this index may be due to the bias in model training for the majority class. Therefore, in this study, we try to use tools and measures that can solve this problem.

**Boxplot:** One of the tools that best represents the center of data aggregation and dispersion.

**G-mean:** A measure that balances the classifier's performance in both the majority and minority classes. The primary purpose of this criterion is to increase the classifier's performance in predicting the positive type. Hence, the small value of this index indicates the model's poor performance in predicting the positive class, even if the negative class is well predicted. This criterion is suitable for preventing the overfitting of the negative class and the poor fit of the positive class.

**Roc Curve:** A popular and widely used graph to show the performance of a binary classification model. It is a probability curve that plots the actual positive ratio against the false-positive ratio at various threshold values.

**AUC:** The area under the ROC curve is often used as a criterion for estimating the prediction of classification models. Its value is between 0 and 1; the larger, the more ideal it is.

**Calibration Plot:** A practical tool for checking the accuracy of the probabilities predicted by the classification model compared to the correct and standard answer. A classifier model has well-calibrated predicted probabilities when actual observed cases coincide with the predicted cases.

**Brier:** Introduced by Brier in 1950 to evaluate meteorological forecasts and passed as a simple scoring rule for evaluating binary model prediction. This index causes discrimination and calibration at the same time. Brier index values, the average prediction squared error, are between 0 and 1, and the smaller the value, the more desirable.

**Delong test**: A widely used nonparametric method for comparing the differences between the performance of two models developed by Delong et al.

**Section 3: An overview of the steps for selecting the optimal feature set for all three methods:**

**scheme of Mutual Information classification feature selection**

1. Select all the features from the original dataset.
2. Determine the relationship between X_i_`s and Y by calculating the mutual information score:

$$MI\left( X | Y \right)=H\left( X \right)-H\left( X | Y \right).$$

$$H\left( X \right), H\left( X | Y \right) are entropy of x and conditional entropy, respectively.$$

3. A higher score indicates the more effectiveness of the X_i_ on Y.
4. Select ten features with the highest mutual information score.

**Scheme of Genetic Algorithm feature selection**

Input D_Tr_: training dataset

Initialize the parameters N=n, t_max_, t = 0;

Ensure: Optimal feature subset with the highest fitness value.

1. While (t≤ t_max_) do
2. Create pop n, t_max_;
3. For k = 1 to n do
   1. Parents [n_1_,n_2_] = selection sheme (n, N)
   2. S= X_OR_[n_1_,n_2_]
   3. Mut= mutation[S]
4. End For
5. Replace n with S_1_, …, S_n_
6. t= t+1;
7. End while
8. Store the highest fitness values;
9. Return the best solution;

**scheme of Random Forest feature selection**

Input A training set R = (x_1_, y_1_), …, (x_n_, y_n_), K features, and number of trees in Forest S

1. Select N trees from the dataset.
2. Construct a decision tree from the N trees.
3. Repeat steps 1 and 2, S times.
4. At each node:
   1. Construct k as a tiny subset of K
   2. Split on best feature in k
5. New records are given to the category that wins the most votes
6. Result: n selected features that have the highest performance.

**Section 4: Optimal parameters:**

The optimal parameters identified by Grid_search are included the parameters of { penalty= 'l2', solver= 'liblinear' }, { kernel='rbf', gamma=0.1 ,C=100,max_iter=-1 }, { criterion='entropy', max depth=10, max features='auto', max leaf nodes=16, min samples split=16,n_estimators = 1000}, and { colsample_bytree=.8,gamma=0,max_depth=9,min_child_weight=1,reg_alpha=1e-05,subsample=.9 } for LR, SVC, RF and XGBoost models respectively.
